# Outbreak of Carbapenem-Resistant High-Risk Clone ST244 of *Pseudomonas aeruginosa* in Dogs and Cats in Algeria

**DOI:** 10.3390/antibiotics14030230

**Published:** 2025-02-24

**Authors:** Amina Badis, Nouzha Heleili, Manel Merradi, Ammar Ayachi, Piera Anna Martino, Gabriele Meroni, Alessio Soggiu

**Affiliations:** 1 ESPA Laboratory, Department of Veterinary Sciences, Institute of Veterinary Sciences and Agronomic Sciences, University of Batna 1, Batna 05000, Algeria; nouzha.heleili@univ-batna.dz (N.H.); m.merradi@univ-batna2.dz (M.M.); 2 Department of Veterinary Sciences, Institute of Veterinary Sciences and Agronomic Sciences, University of Batna 1, Batna 05000, Algeria; aayachi54@yahoo.fr; 3 Department of Microbiology and Biochemistry, Faculty of Natural and Life Sciences, University of Batna 2, Batna 05078, Algeria; 4 One Health Unit, Department of Biomedical, Surgical and Dental Sciences, University of Milan, 20122 Milan, Italy; piera.martino@unimi.it (P.A.M.); alessio.soggiu@unimi.it (A.S.); 5 Fondazione IRCCS Ca’ Granda Ospedale Maggiore Policlinico, 20122 Milan, Italy

**Keywords:** *Pseudomonas aeruginosa*, dogs, cats, carbapenem resistance, ST244, virulence, biofilm, Algeria, whole genome sequencing, MinION

## Abstract

**Background/Objectives: ***Pseudomonas aeruginosa* causes chronic infections in humans and animals, especially cats and dogs. This bacterium’s ability to adapt and acquire antibiotic resistance traits may complicate and exacerbate antibacterial therapy. This study aimed to evaluate the antibiotic resistance patterns, virulence factors and ability to form biofilms of *P. aeruginosa* strains isolated from Algerian dogs and cats. **Methods**: Nineteen samples were collected from healthy and diseased dogs and cats. Isolates were studied for their antibiotic-resistance patterns (disc diffusion method) and biofilm formation (Microtiter assay) and were whole-genome sequenced (MinION). **Results**: Nineteen *P. aeruginosa* strains (15 from dogs and 4 from cats) were isolated. Antibiotic-resistance phenotypes were observed against amoxicillin–clavulanic acid (100%); meanwhile, resistance towards ticarcillin was 40% (dogs) and 25% (cats), ticarcillin–clavulanic acid was 13.33% and 25% for dogs and cats, respectively, and imipenem was 75% (cats) and 20% (dogs). Moreover, 95% of strains were biofilm-producers. Different antimicrobial resistance genes (ARGs) were found: beta-lactamase genes, mainly *PAO*, *OXA-494*, *OXA-50* and *OXA-396* and an aminoglycoside gene (*aph(3′)-IIb*). The main high-risk sequence types (STs) were ST244, 2788, 388 and 1247. A large panel of virulence genes was detected: *exoS*, *exoT*, *exoY*, *lasA*, *toxA*, *prpL*, *algD*, *rhIA* and others. **Conclusions**: The genetic variety in antibiotic-resistance genes of resistant and virulent *P. aeruginosa* strains in dogs makes public health protection difficult. Continuous monitoring and research in compliance with the One Health policy are needed to solve this problem.

## 1. Introduction

*Pseudomonas aeruginosa* is an important Gram-negative opportunistic pathogen of humans and animals [1]. This bacterium can cause various infections in dogs, including ulcerative keratitis, otitis, pyoderma, urinary tract infections, skin infections, wound infections and respiratory tract infections [2]. Infections caused by *P. aeruginosa* may be linked to immunosuppression in companion animals, including documented cases in dog post-kidney transplantation [3] and in connection with cancer treatments [4]. Moreover, it acts as a pathogen in cats, although it is less common than in dogs [5,6], and the reported infections include respiratory tract infections [3,7], as well as ulcerative keratitis and wound infections [8].

Due to its increased antibiotic resistance, the World Health Organization (WHO) has classified carbapenem-resistant *P. aeruginosa* as a high-priority bacterium. It was designated as one of the ESKAPE pathogens (*Enterococcus faecium*, *Staphylococcus aureus*, *Klebsiella pneumoniae*, *Acinetobacter baumannii*, *Pseudomonas aeruginosa* and *Enterobacter* species) because of its capacity to escape killing by acquiring exogenous genes and developing resistance through a variety of internal pathways, which promotes the emergence of multidrug resistant strains [9,10]. The European Food Safety Authority’s Panel on Animal Health and Welfare has classified *P. aeruginosa* as one of the most significant antimicrobial-resistant bacteria in the European Union (EU), with over 90% certainty [11].

*P. aeruginosa* infections are worrying because their treatment poses a major global challenge due to the development of resistant strains in humans and animals. Antibiotics have become less effective or even ineffective against this bacterium due to its resistance mechanisms, leading to therapeutic failure. The main resistance mechanisms are beta-lactamase production, efflux pumps, induced mutations and biofilm production [12]. Some authors reported that *P. aeruginosa* strains showed a high resistance rate to multiple antimicrobial agents [13]. Its infections in pets are currently generally treated with broad-spectrum antibiotics [14], and close interactions between pets and humans make significant opportunities for the interspecies transmission of resistant bacteria and horizontal transfer of antibiotic resistance genes in both directions, mainly through physical injuries, petting or licking activities [15]. *P. aeruginosa* is also recognized for its ability to quickly acquire additional resistances, meaning that the combination of intrinsic and acquired resistance can result in therapeutic failures [16].

Biofilm formation is a crucial survival strategy used by *P. aeruginosa* to endure challenging conditions, such as exposure to antibiotics and host immune defenses [17]. Moreover, it displays various virulence factors, including exotoxins (*toxA*, *toxR*), elastases (*lasB*), proteases (*plcH*) and alginate (*algD*), all of which contribute to the development of severe diseases. The type 3 secretion system (T3SS) is a significant virulence factor, which delivers four cytotoxins, including *exoU* [18].

There are few reports documenting the patterns of antimicrobial resistance and virulence factors of *P. aeruginosa* isolated from companion animals, and until now, there are no studies focused on the epidemiology of this pathogen in Algeria. The study aimed to investigate the antimicrobial-resistance profiles and the associated resistance and virulence genes of *Pseudomonas aeruginosa* strains isolated from dogs and cats in some regions of Eastern Algeria. As the first study in Algeria, it aimed to provide valuable insights into the epidemiological characteristics and potential health risks of these strains in companion animals, highlighting their implications for veterinary and public health.

## 2. Results

### 2.1. General Population Information

Table 1 summarizes the basic information of each analyzed *P. aeruginosa* strain. Fifteen were isolated from dogs, mostly from nasal swabs (n = 6), followed by the rectum (n = 5) and middle ear (n = 4). Four cats were positive for *P. aeruginosa* from the rectum. One isolate was collected per animal.

### 2.2. Antimicrobial Susceptibility Testing

Table 2 displays the prevalence of resistance to each antimicrobial agent. All the strains were resistant to amoxicillin–clavulanic acid (AMC) in both cats and dogs, and 36.8% and 15.8% of the strains were resistant to ticarcillin (TC) and ticarcillin–clavulanic acid (TCC) from dogs (40%) and cats (25%), respectively. The resistance rate to imipenem was 36.8%, with 20% in dogs and 75% in cats. No resistance towards aminoglycosides and fluoroquinolones was detected. None of the isolates were multidrug resistant.

### 2.3. Biofilm Formation

Diverse biofilm profiles were identified; 42% (n = 8) were classified as strong biofilm producers, 25% (n = 5) as moderate producers, all isolated from dogs, and 27% (n = 5) as weak producers, including four isolates from cats and one from a dog. One strain, a non-producer, was identified from a dog. All isolates identified as strong producers were derived from dogs; 37.5% were isolated from the rectum, with an equal number from the nasal cavity. Additionally, 60% of dog strains exhibiting moderate production were isolated from the nasal cavity. There was no relation between the sampling site and the ability of biofilm production (*p* = 0.430).

### 2.4. General Features of the Genomes

All the strains were uploaded to NCBI database under Bioproject PRJNA1153397. After genome assembly, the sequencing data from the Nanopore Mk1C includes a mean coverage of 120× with an N50 of 6,216,739 bp and a genome size of 6,560,953.684 bp. Table 3 summarizes the main statistics of the genomes assembled and polished.

**Figure 1:**
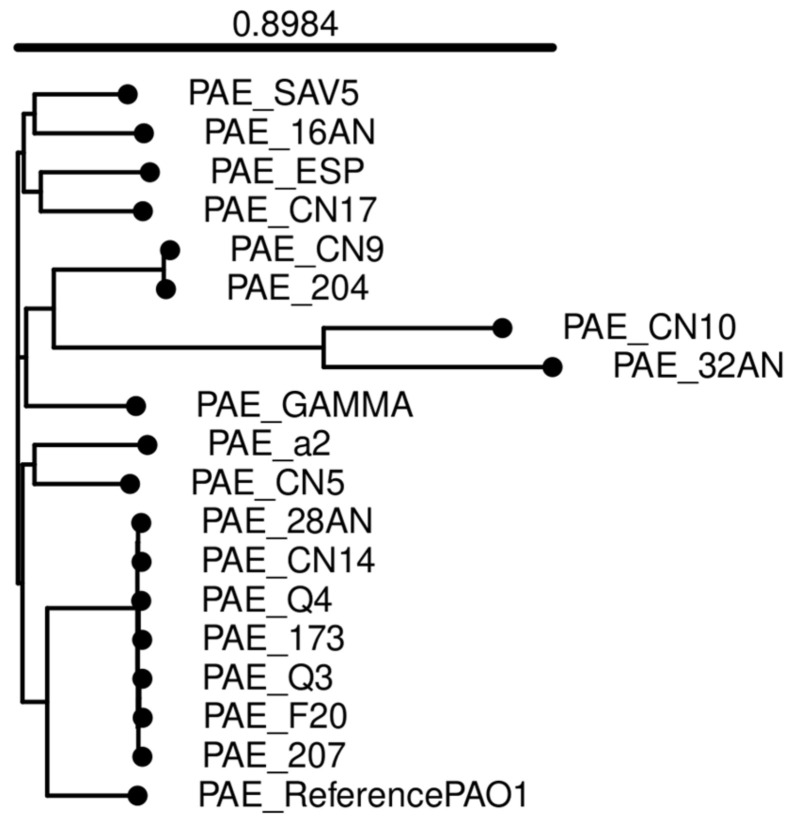
Whole-genome-based phylogenetic tree built with IPGA.

A whole-genome based phylogenetic tree (Figure 1) was built with the online tool Integrated Prokaryotes Genome and pan-genome Analysis service IPGA (v1.09) (accessed on 13 June 2024), including the reference strain (PAO1) used during the bioinformatic analyses [19].

**Figure 2:**
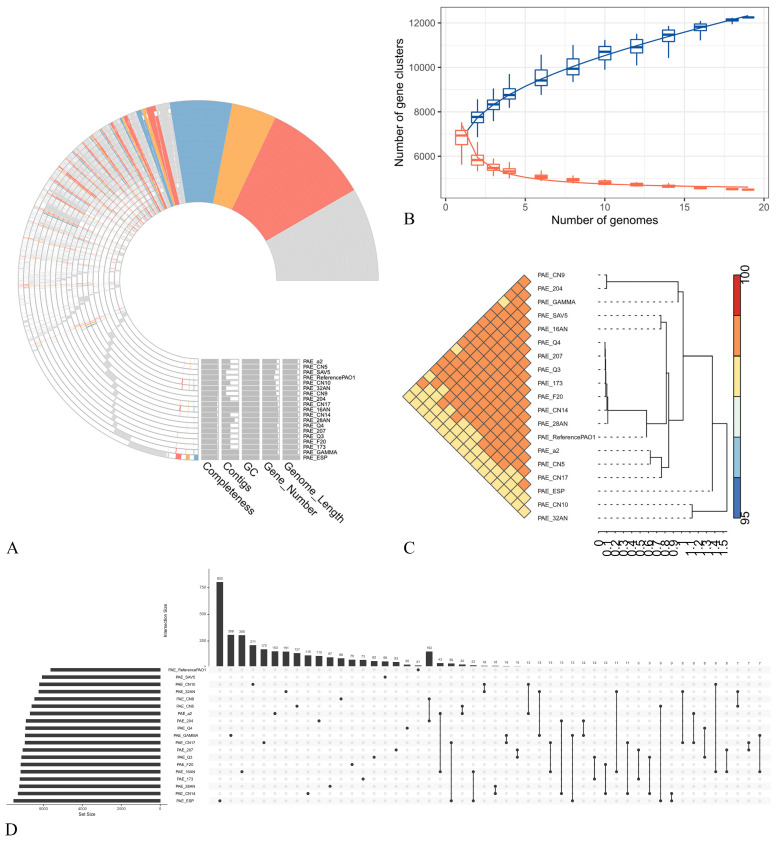
Pan-genome analysis of *P. aeruginosa* strains. (**A**) Pan-genome profile. (**B**) Number of pan-gene clusters (blue) and core gene clusters (orange) among the isolates. (**C**) Heatmap and hierarchical clustering based on pairwise average nucleotide identity (ANI) values. (**D**) Upset plot of comparisons among unique genes of strains.

**Figure 3:**
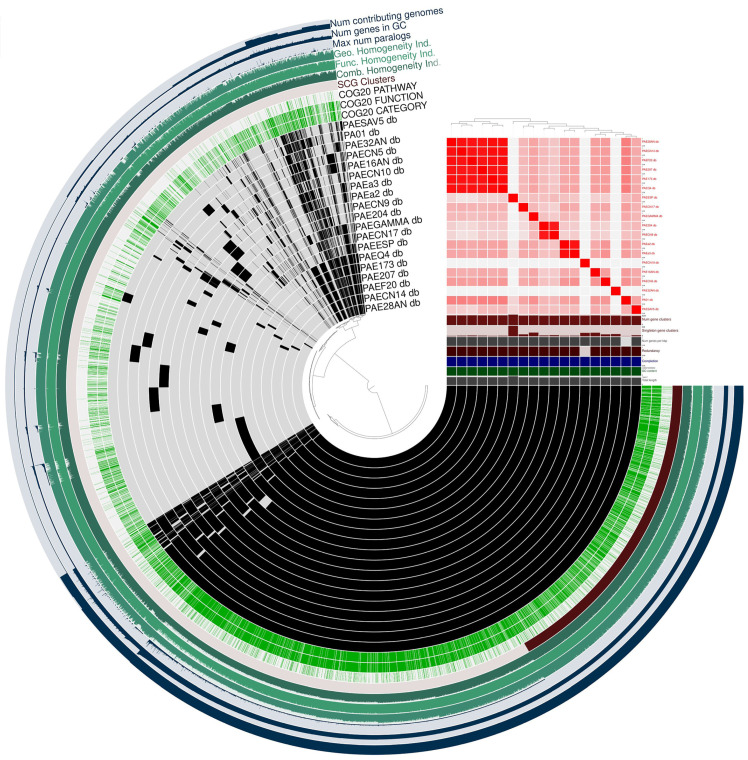
Pangenome visualization of analyzed *P. aeruginosa* strains. The pangenome analyses were visualized using ANVI’O (v8). The central dendrogram grouping the samples is arranged according to the presence or absence of gene clusters. Order of items: count of genomes for which each gene cluster contains matches. The phylogenetic tree displays the samples arranged according to the ANI % identity. Each sample cluster is denoted by a red square in the phylogenetic tree, indicating ANI percentage identity values over 99%.

The pan-genome profile of all the *P. aeruginosa* strains is reported in Figure 2A. Red represents metabolism genes, orange represents those related to information storage and processing, while genes involved in cellular processes and signaling are blue, and finally, grey genes are poorly characterized or unannotated genes. Together, these components constitute the *core* genes shared between the analyzed strains. As shown in Figure 2B, 12,250 pan-gene clusters were identified. The average nucleotide identity (ANI) analysis (Figure 2C) revealed a high (>98%) identity among all the strains. The upset plot (Figure 2D) indicated that the distinct gene clusters in each genome ranged from 15 to 803. The pan-genome profile derived from Clusters of Orthologous Genes (COG) annotation indicated that the *core* gene clusters comprised 2480 for metabolism, 1102 for information storage and processing, 1521 for cellular functions and signaling and 718 were poorly characterized or unannotated. Figure 3 represents the pangenomes visualized using ANVI’O to address various aspects of interactive displays.

### 2.5. Multilocus Sequence Typing (MLST)

**Figure 4:**
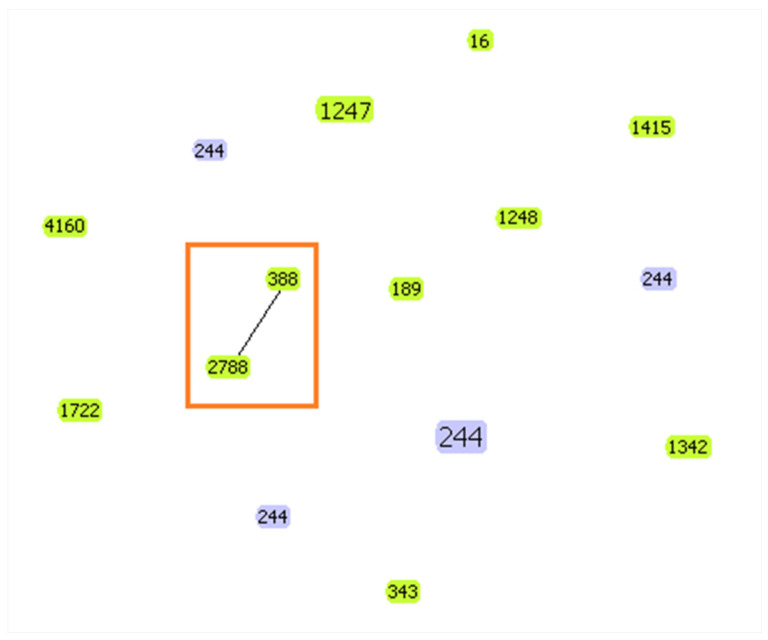
Clonal complex and singletons obtained.

MLST analysis revealed 12 different sequence types (ST4160, ST1248, ST1247, ST2788, ST1722, ST189, ST343, ST16, ST1415, ST388, ST244, ST1342). The predominant ST was the high-risk clone ST244 found in seven strains (two from cats and five from dogs) first described in pets in Algeria. These strains were isolated from different sampling sites, the rectum in cats and the rectum, ear and nasal cavity in dogs. On the other hand, two other sequence types were isolated from cats (ST1247 and ST1342), and the other STs were recovered from dogs. One clonal complex (Figure 4) was displayed, comprising two STs (2788 and 388) with a double *loci* variant, and the rest, which did not share at least two of the seven loci, were considered singleton STs. The isolates from the same ST are clustered according to the phylogenetic tree. Figure 4 was generated using the geoBURST software (v1.2.1) [20]. Supplementary Table S1 reports the ST distribution among the geographical sampling sites.

### 2.6. Antibiotic Resistance and Virulence Genes

**Figure 5:**
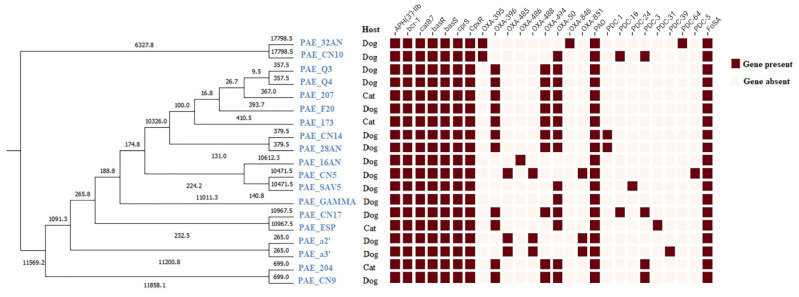
Phylogenetic tree and distribution of resistance genes among analyzed strains.

Seventy-six resistance genes were found in *P. aeruginosa* genomes (Figure 5). Most of them were associated with the efflux pump systems. The genes *aph(3′)IIb* and *bcr-1*, conferring resistance to aminoglycosides and bicyclomicin, were present in all isolates. Furthermore, the peptide resistance genes *basR*, *basS*, *cprS* and *cpxR* were also identified in all the isolates.

The results from WGS showed that all the strains harbored at least two genes responsible for beta-lactam resistance. The predominant gene was *bla_PAO_* found in all isolates, followed by *bla_OXA-50_* and bla_*OXA-396*_ in 73.7% and 57.9% of the strains, respectively.

All the strains resistant to imipenem harbored the following genes: *OXA_396_*, *OXA_494_*, *OXA_50_* and *bla_PAO_*. The combination between *bla_OXA_* and *bla_PDC_* was found in 57.9% of the strains; 84.2% of the strains harbored more than three genes responsible for beta-lactam resistance. The aminoglycoside-resistance gene *aph(3′)IIb* was detected in all the isolates. At least one of the *bla_PDC_* variants was present in 11 strains (57.9%).

**Figure 6:**
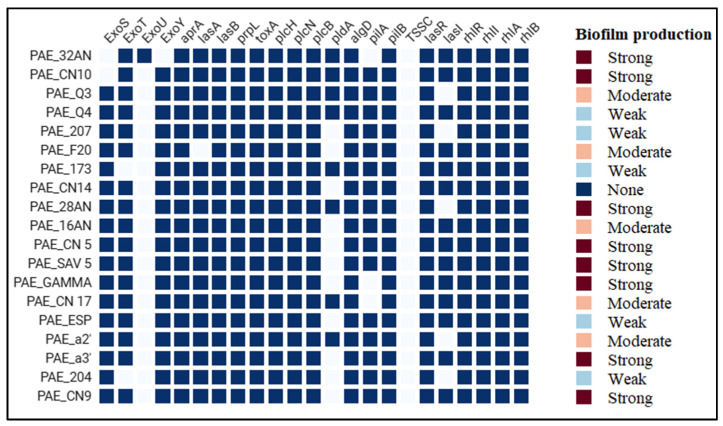
Distribution of the virulence genes and the biofilm-production ability.

All genomes were screened for virulence factors (Figure 6), resulting in the identification of those responsible for motility and adhesion, *quorum*-*sensing*, biofilm production, the type III secretion system, siderophore production, proteases, toxins and enzymes.

Eighty genes associated with adherence and motility were detected, with variations observed in their distributions among the isolates. All of them contained the gene essential for lipopolysaccharide (LPS) production. Of the 46 genes involved in flagellar assembly, 35 were present across all strains, with *fleS* gene being the most frequently absent, missing in 63.2% of the genomes. Regarding the genes responsible for type IV *pili* biosynthesis, *pilA* and *pilB* were found in 84.2% and 100% of the strains, respectively.

The frequency of *exoU* and *exoS* was 5.3% (1/19) and 89.5% (17/19), respectively.

WGS analysis revealed that the predominant type three secretion system (T3SS) virulotypes were *exoU*−/*exoS*+ found in 17 strains (89.5%), *exoU*+/*exoS*− and *exoU*−/*exoS*− in one isolate each.

Proteases, toxins, and enzymes are among the most effective virulence factors contributing to the severity of *P. aeruginosa* infection. All the isolates harbored the genes *aprA*, *lasB* and *prpL*, coding for proteases. The *toxA* gene, coding for the exotoxin A, was also present in all the isolates. Regarding genes encoding enzymes, *plcH*, *plcN* and *plcB* were present in all isolates, while *pldA* was found in only eight isolates (42.1%)

Additionally, the isolated *P. aeruginosa* strains exhibited a 100% prevalence of the *quorum-sensing* genes *lasR*, *rhlR* and *rhlI*, while the *lasI* gene was identified in 73.7% of the strains.

Alginate and lipopolysaccharide are essential for biofilm formation, and the genes encoding their synthesis, *algD* and *LpS*, were found in all the strains.

## 3. Discussion

*P. aeruginosa* is a significant opportunistic and challenging-to-treat bacterium. It is commonly present in various environments, including dogs and cats, though it is not considered part of their normal flora [21]. Healthy animals are less likely to develop *P. aeruginosa*-related diseases, but the bacterium can colonize the skin and mucous membranes without causing an infection [22]. Several predisposing factors can increase the risk of infection in dogs, such as existing health issues, environmental stressors and anatomical factors (like ear canal structure in certain breeds), promoting bacterial growth and leading to conditions like otitis [23]. This pathogen aligns with the concept of “One Health” [24] due to its unique characteristics, including extensive environmental diffusion [25], intrinsic resistance to several classes of antibiotics [26], high capacity to acquire new resistance mechanisms [27] and numerous virulence factors [28].

In the present study, *P. aeruginosa* was isolated at a low prevalence of 7.5% and 1.91%, respectively, from the middle ear, rectum and nasal cavities of canines and cats. These rates are lower than those documented in an earlier investigation in Thailand by [29], at 79.6% and 20.4%. In dogs, our results are similar to previous reports in Italy (8%) [27] and China (6.7%) [30]. However, the prevalence is higher than earlier reports in India and California (3% each) [31,32]. Other studies conducted in Romania (40.84%) [33], South Korea (18.75%) [34] and Brazil (31.62%) [35] had reported higher prevalence rates than our findings. In cats, we observed a prevalence of 1.9%, close to the results reported by Gentilini et al. (2018) in Italy at 1.41% [36].

With no MDR or XDR isolates, the antibiotic-resistance profile of the isolates from this study was lower than that of academic research on *P. aeruginosa* isolated from dogs and cats. Research in Tunisia revealed that all isolates were susceptible to all antibiotics [37], despite other studies from humans and animals [38] reporting significant antibiotic resistance in *P. aeruginosa* isolates. According to Valero et al. (2019), the beta-lactams ceftazidime, cefepime and piperacillin/tazobactam demonstrated susceptibilities greater than 85% [39]. In a similar study in Romania, strains isolated from dogs with superficial skin infections showed high resistance rates to aminoglycosides (62.06% for CN, 55.17% for AK and 91.37% for TOB) [33]. In contrast, our findings showed no resistance to this class of antibiotics. In our study, no resistance to ciprofloxacin was detected, which contrasts the findings of Feßler et al. (2022), who reported a resistance rate of 16.1% in dog isolates, while no resistance was observed in cats, thus aligning with our findings [40]. Rubin et al. (2008) reported that ciprofloxacin was the most effective fluoroquinolone labeled for veterinary use, with 16% of canine-resistant *P. aeruginosa* [41].

Imipenem-resistant strains have been identified with a frequency of 20% in dogs and 75% in cats, indicating a significant public health risk. This class of antimicrobials, a key class in human medicine, is not used for treating animal infections nor is it licensed for veterinary use [42]. Carbapenem resistance was not extensively studied; several reports have indicated a high resistance prevalence [11,43,44]. In Algeria, this study is the first report of carbapenem-resistant *P. aeruginosa* isolated from pets, while other Algerian reports focused on carbapenem-resistant Enterobacterales in companion animals [45,46]. The emergence of such high resistance levels has been documented in over 25 reports on dogs and cats published worldwide (da Silva et al., 2022). This could severely limit therapeutic options and complicate infection management in both animals and potentially humans [11,47]. Notably, our results show significantly higher resistance rates than those reported by [48] in Japan, where only 3.4% of strains exhibited resistance to imipenem.

Nevertheless, our results are approximately similar to those found by [49] at 30%, while [37,50] found one carbapenem-resistant isolate among 181 *P. aeruginosa* strains in Portugal and 66 in Tunisia, respectively. This discrepancy in resistance may be attributed to the sampling site, the overall health condition of the animals and the impact of antibiotic use as a key contributing factor; the use of carbapenems is limited to treating urinary and respiratory tract infections in cats and dogs [51], making it likely that Algerian veterinarians have depended on this class of antibiotics for management. The isolation of carbapenem-resistant strains in this context from non-human sources is of a great risk to public health, and their origin can be the human–pet bond [51].

MLST is an excellent tool for long-term, worldwide epidemiological research. In our study, we identified 12 STs from dogs and cats, each from a different strain, while ST244 was found in eight strains, and one clonal complex was detected (2788 and 388). A clonal complex indicates a strong resistance-related genetic association between the current isolates [48]. This can affect bacterial resistance, as closely related strains may harbor shared resistance genes or protective mechanisms against antibiotics. The CC244 clonal complex was detected in *P. aeruginosa* in pediatric populations in China with ST244, ST8818, ST1701 and ST1103. Isolates belonging to CC244 demonstrated significantly higher resistance [52].

ST244 is significant for its role in the horizontal acquisition of antibiotic-resistance genes through mobile genetic elements [53] and is recognized as fourth among the top ten high-risk *P. aeruginosa* epidemic lineages worldwide [54] associated with MDR [55]. This epidemic clone is one the most extensively researched clones, as mentioned in 182 articles, highlighting its significance in disseminating antimicrobial-resistance genes (ARGs) [53]. The global spread of high-risk *P. aeruginosa* clones has posed a significant public health challenge since their microevolution in aggressive environmental conditions by acquiring new mutations in their genome, leading to new antimicrobial resistances [56]. In addition, many difficult-to-treat infections resulted from high-risk clone strains with higher pathogenicity and virulence levels and an increased capacity to colonize and persist within a host [57]. However, our strains did not show multidrug-resistance phenotypes.

ST244, the second most prevalent Mediterranean *P. aeruginosa* clone, was frequently reported in study [58] in hospitals in Annaba and Skikda in northeastern Algeria and by [58] in Batna hospital in eastern Algeria. ST244 is one of the most prevalent high-risk genotypes in Europe in epidemics [59]. Since ST244 was recovered from both dogs and cats and *P. aeruginosa* is considered an important source of community and hospital-acquired infections, these community-acquired *P. aeruginosa* strains may be more prone to disseminate in the surrounding environment. Our study, which was the first conducted in Algeria, examined the sequencing of *P. aeruginosa* in companion animals, making it impossible for us to compare our results with those of previous research. The level of antibiotic resistance found in some strains could be interpreted as human-derived mainly for clones belonging to ST244, ST1248, ST1342 and ST16 (originally described in humans [60,61,62,63]) that could be transmitted to companion animals [21,64]. This condition could lead to a genetic rearrangement to adapt to the new host with the surrounding environmental and antibiotic pressure.

In this study, we performed WGS to detect resistance and virulence-associated genes. The detected ARGs *aph(3′)-IIb*, *fosA* and *catB7* are documented worldwide in *P. aeruginosa* and often located on the chromosome [65], underlining the intrinsic resistance of the bacterium to phenicols and fosfomycin. The *bla_PAO_* gene is a cephalosporinase encoded in the chromosome and in *P. aeruginosa* is prevalent among multidrug-resistant strains [66].

Similarly to the ARGs detected in our strains conferring resistance to beta-lactams and aminoglycosides, the complete genome sequence of *P. aeruginosa* strains from a canine skin lesion showed the presence of *aph(3′)-IIb*, *bla_OXA-488_* and *bla_PAO_* as ARGs [1] and *bla_PAO_*, *bla_PDC-24_*, *bla_OXA-486_* and *aph (3′)-IIb* in a carbapenem-resistant *P. aeruginosa* isolate from red deer [50]. Also, a multidrug-resistant *P. aeruginosa* isolate from a dairy cow with chronic mastitis carried *bla_OXA-485_*, *bla_OXA-488_*, *aph(3′)-IIb* and *bla_PAO_* [67]. *bla_PAO_* and *bla_OXA50_* presented high prevalence in the *P. aeruginosa* genome [68].

Whole-genome sequencing of *P. aeruginosa* (Figure 2) identified many antibiotic-resistance gene sequences, suggesting that this bacterium possesses the ability for spontaneous transformation, as suggested from the literature [69].

Regarding the virulence genes, the transcription of many genes is controlled by a mechanism known as *quorum*-*sensing* (QS). The *lasR*, *rhlR* and *rhlI* genes were identified in all the strains (100%), unlike *lasI*, found in 73.7% of the strains. Therefore, 73.7% of the strains harbored all the genes of QS together.

The high prevalence of the *exoS*, *exoT* and *exoY* genes in the present study (89.5%, 89.5% and 94.7%, respectively) was consistent with the existing literature [49,70]. The most common virulotype identified in our study was *exoU*−/*exoS*+ (89.5%), while the least frequent was *exoU*+/*exoS*+, which was absent in all the strains. These findings are similar to those that Hayashi et al. (2021) reported, with respective rates of 81.3% and 1.3% [70]. These type three secretion system effectors are crucial contributors to mortality [71]. Strains with both *exoU* and *exoS* cytotoxins have been found in other studies [72]. *exoT* is the most frequent effector in genomes of clinical and environmental *P. aeruginosa* [73,74]. The *ExoY* gene was predominant in the genomes of both urinary and environmental strains [75].

In agreement with our results, the genes *toxA*, *lasB* and *plcH* were identified in all strains, confirming the findings reported in the literature [27,76]. Similarly, in line with our findings, the gene coding for alkaline protease *aprA*, was found in all strains, while the *toxA* gene was present in 91.7%, confirming previous results [27].

The *algD* gene encodes, an enzyme that synthesizes the polysaccharide alginate, an important component of the biofilm produced by *P. aeruginosa*, was identified in all the strains. Our finding showed that 95% of all isolates could form a biofilm. According to [24], biofilm-forming strains comprised 90.6% of *P. aeruginosa* isolates from dogs and 86.4% from cats. Of these biofilm-forming bacteria, 26.3% had poor, 35.0% had intermediate and 38.7% had strong biofilm formation. However, a study conducted in Portugal, in which the biofilm-forming ability of *P. aeruginosa* isolated from dogs was investigated, found that all the isolates appeared to be weak biofilm producers [49], and in another study conducted by Hattab et al. (2021), among canine isolates, five isolates (20.8%) were classified as strong biofilm producers, while 8 (33.3%) and 11 (45.8%) isolates were weak and intermediate biofilm producers, respectively [27]. The *algD* operon encodes the enzymes required for alginate production. The expression is controlled by many factors, including the alternative sigma factor *AlgU* (σ^22), which activates transcription of the *algD* promoter. *AlgU* is triggered under stressful circumstances (e.g., during infection). This control is essential for the change from a non-mucoid to a mucoid phenotype, as seen in *P. aeruginosa* strains obtained from cystic fibrosis patients [77]. WGS analysis identified alternative genes implicated in biofilm production (via *quorum-sensing* pathway) apart from *algD*; this is the case for the *las* and *rhl* operons [78]. All the strains, even the one that phenotypically could not form a biofilm, carried *lasA*,*B*,*I* and *rhlA*,*B*,*C*,*L* genes.

## 4. Materials and Methods

### 4.1. Population Study and Sample Collection

**Figure 7:**
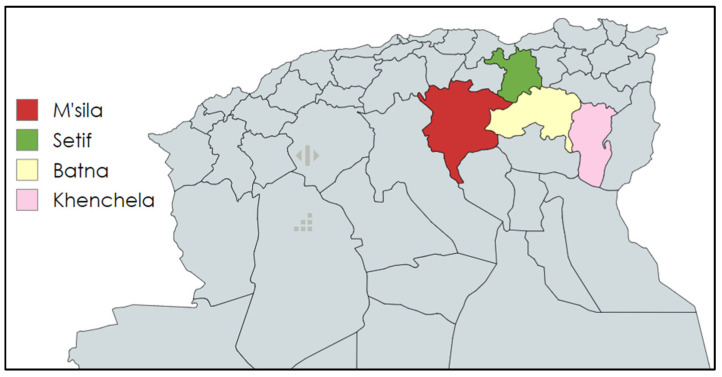
Map of northern Algeria highlighting the sampling areas (https://www.mapchart.net/, accessed on 24 September 2024).

From February 2021 to December 2023, 409 samples were taken from healthy and clinically ill dogs and cats from different breeds in various regions of eastern Algeria: Batna (n = 281), Khenchela (n = 58), Setif (n = 66) and M’sila (n = 4) (Figure 7). For cats, samples were collected from the rectum (n = 102), ear (n = 30), abscesses (n = 29), wounds (n = 17), the uterus (n = 12), the nasal cavity (n = 10), urine (n = 1), the buccal cavity (n = 8) and 4 of another origin. In dogs, the sample sites included the ear (n = 88), nasal cavity (n = 70), rectum (n = 25), wound (n = 6), abscesses (n = 4), eye (n = 2) and vagina (n = 3). After sampling, the samples were refrigerated and shipped to the laboratory for bacterial investigation.

This study was conducted following the requirements of the Scientific committee of the Institute of Veterinary and Agricultural Sciences (University of Batna 1), under the certificate of animal use Protocol N°: 001/DV/ISVSA/UB1/2025.

### 4.2. Culture Conditions and Bacterial Identification

Upon arrival at the laboratory, swabs were incubated in BHIB (Brain Heart Infusion broth; Himedia, Nashik, India) for 24 h at 37 °C and then cultivated aerobically on cetrimide agar (Merck, Darmstadt, Germany) at 42 °C for 24–48 h. Culture is considered positive when greenish colonies, oxidase- and catalase-positive, and Gram-negative rods (after Gram staining) are observed. Cultures were then purified on MacConkey agar (Merck, Darmstadt, Germany), and pure strains were identified using the API 20 NE system^®^ (Biomérieux, Craponne, France).

### 4.3. Antimicrobial Susceptibility Testing

Antimicrobial susceptibility testing was performed using the Kirby–Bauer disc diffusion method according to the EUCAST guidelines [79]. Bacterial suspensions were adjusted to the 0.5 McFarland turbidity standard. Bacterial isolates were tested based on a panel of 13 antibiotics: amikacin (AK) 30 µg, ceftazidime (CAZ) 30 µg (Oxoid, Basingstoke, UK), gentamicin (CN) 15 µg (Bioanalyse, Ankara, Turkey) netilmicin (NET) 30 µg, tobramycin (TOB) 10 µg, levofloxacin (LEV) 50 µg, ciprofloxacin (CIP) 5 µg, cefepime (FEP) 30 µg, aztreonam (ATM) 30 µg, ticarcillin (TC) 75 µg, ticarcillin–clavulanic acid (TCC) 75/10 µg, imipenem (IMP) 10 µg and amoxicillin–clavulanic acid (AMC) 20/10 µg (Biomaxima, Lublin, Poland). *P. aeruginosa* ATCC 27853 was used as a quality control strain.

### 4.4. In Vitro Biofilm-Formation Assay

To study the biofilm-formation ability, the microtiter assay was performed, using a protocol previously described [80]. BHIB broth induced biofilm formation, and *P. aeruginosa* ATCC 27853 was used as a positive biofilm producer.

Bacterial optical densities were measured using a microplate reader (M 960, Metertech, Taipei, China), at 550 nm using a 30% (*v*/*v*) solution of acetic acid as the blank. The optical density of each isolate (A_550_) was obtained by averaging 8 wells and then compared with the cutoff value (0.102), which was determined arbitrarily by the mean of the negative control (0.042) plus three standard deviations (0.02). The level of biofilm production was classified into four categories: no production A_550_ < 0.101, weak biofilm production 0.101 ≤ A_550_ < 0.202 (2× negative control), moderate biofilm production 0.202 ≤ A_550_ < 0.408 (4× negative control) and strong biofilm production 0.404 < A_550_, according to the literature [24].

### 4.5. Whole-Genome Sequencing (WGS) and Bioinformatics Analysis

#### 4.5.1. DNA Extraction

All *P. aeruginosa* strains were sequenced using a long-read sequencing (LRS) approach based on the MinION Mk1C platform (Oxford Nanopores Technologies, Oxford, UK). Isolated colonies from a fresh culture of *P. aeruginosa* on blood agar were resuspended in 500 µL of PBS (Phosphate Buffered Saline, Euroclone, Milan, Italy) and centrifuged at 10,000× *g* for 1 min at room temperature, and high-molecular-weight DNA was extracted with the Quick-DNA^TM^ HMW MagBead Kit (Zymo Research, Irvine, CA, USA) following the manufacturer’s instructions. The DNA quantity and quality were assessed using a NanoReady Touch series Micro Volume (UV-Vis) (Aurogene, Roma, Italy), ensuring that the A_260_/A_280_ and A_260_/A_230_ ratios ranged between 1.8 and 2, respectively. Extracted DNA was also subjected to gel electrophoresis to check its integrity.

#### 4.5.2. Whole-Genome Sequencing

The sequencing libraries were prepared with 200 ng, as input DNA, and they were subjected to transposase fragmentation with the Rapid Barcoding Sequencing kit (SQK-RBK114.24, Oxford Nanopore Technologies, Oxford, UK). Then, 12 isolates were multiplexed on a single flow cell (FLO-MIN114, R10.4.1 version) and sequenced in a MinION Mk1C for 72 h maximum.

#### 4.5.3. Bioinformatic Analysis

Dorado (v0.8.2) was used to basecall (--dna_r10.4.1_e8.2_400bps_hac@v5.0.0), trim adapters and demultiplex .pod5 files (--config configuration.cfg --barcode_kits SQK-RBK114.24 --trim_barcodes; min_score threshold default 60). Summary statistics were obtained with NanoPlot (v1.44.0) (--verbose --tsv_stats –N50 --fastq) [81]. Reference guided filtration with a 1000 bp threshold was achieved using FiltLong (v0.2.1), blasting each strain against *Pseudomonas aeruginosa* reference strains PAO1 (--assembly Ref_PAO1.fasta --trim --min_length 1000 --keep_percent 90) [82]. Genomes were de novo assembled using Flye (v2.8.1-b1676) (--nano-corr --genome-size 5m --asm-coverage 50 --plasmids --trestle) [83]. Assembled contigs were polished with Medaka (v2.0.1) (medaka_consensus --t 8 --m dna_r10.4.1_e8.2_400bps_hac@v4.1.0) [84]. Genomic completeness and contamination were derived using CheckM2 (v1.0.2) (checkm2 predict --threads 30 –x fna) [85].

The NCBI Prokaryotic Genome Annotation Pipeline (PGAP) was used to annotate genomes and find out the total numbers of coding sequences, rRNAs and tRNAs [86].

Multilocus sequence types (MLSTs) were determined by uploading the genomes to the Center for Genomic Epidemiology (https://www.genomicepidemiology.org/, accessed on 16 January 2025) using the online tool for MLST prediction, coupled with the PubMLST website (https://pubmlst.org/organisms/pseudomonas-aeruginosa, accessed on 16 January 2025). eBURST software was used to visualize clonal complexes [20]. Finally, pan-genomes were visualized using both an online tool (IPGA) [19] and a command-line pipeline (ANVI’O) [87].

#### 4.5.4. Bioinformatic Analysis of Antimicrobial-Resistance Genes and Virulence Factors

To ascertain the presence of antibiotic-resistance genes (ARGs), the Comprehensive Antibiotic Resistance Database (CARD), the National Centre for Biotechnology Information (NCBI), ResFinder, Plasmidfinder and the virulence factor databases (all updated on 4 November, 2023) were used in the analysis of genomes using Abricate (v1.0.1) (abricate/path/to/fna/*.fna --db card, vfdb, resfinder, ncbi, plasmidfinder --minid 95 --csv >/path/to/output/.csv) [88,89,90,91,92,93,94,95]. For all of these analyses, a threshold identity ≥ 95% was set.

### 4.6. Statistical Analysis

To analyze the associations in the distribution of *P. aeruginosa* strains across sample origins and for the antibiotic phenotype comparison, a chi-squared test (Chi^2^) and Fisher’s test were performed at a 95% confidence interval (α = 0.05) to assess if observed species distributions differed significantly across origins. All statistical analyses in this study were performed with SPSS (v26.0.0).

## 5. Conclusions

This study investigated the resistance patterns of *P. aeruginosa* isolates from cats and dogs. In Algeria, this is the first identification of carbapenem-resistant *P. aeruginosa* (31.56%) in pets with an arsenal of resistance genes, mainly those related to aminoglycosides (*Aph(3′)IIb*), beta-lactams (*bla_OXA_*, *bla_PAO_*, and *bla_PDC_*) and bicyclomicin (*bcr-1*) resistance, with the emergence of high-risk clone ST244. A high capacity for biofilm production (42% strong producers) and a wide range of virulence genes were associated with the third system of secretion, *quorum-sensing* and others. The research underscores the need to comprehend *P. aeruginosa* resistance patterns across diverse populations and areas, promoting judicious antibiotic utilization and stringent infection control measures to avert the dissemination of resistant strains. Furthermore, monitoring antibiotic resistance in companion animals should be intensified to prevent the potential transmission of infections between animals and their owners.

## Figures and Tables

**Table 1:** Distribution of the strains among the pets.

Isolate ID	Animal	Sex	Breed	Age (Month)	Sampling Source	Health Status	Geographical Location
PAE_32AN	dog	M	German shepherd	3	nasal cavity	H	Setif
PAE_CN10	dog	M	na	na	rectum	H	Batna
PAE_PAECN9	dog	M	na	na	rectum	H	Batna
PAE_a3	dog	F	German shepherd	4	middle ear	H	Khenchela
PAE_16AN	dog	M	German shepherd	6	nasal cavity	H	Setif
PAE_CN5	dog	M	na	12	rectum	H	Batna
PAE_GAMMA	dog	M	Malinois	12	nasal cavity	H	M’sila
PAE_SAV5	dog	M	German shepherd	48	middle ear	D (otitis)	Batna
PAE_Q4	dog	M	Malinois	18	middle ear	H	Khenchela
PAE_Q3	dog	M	Malinois	18	nasal cavity	H	Khenchela
PAE_CN17	dog	F	na	na	rectum	H	Batna
PAE_a2′	dog	M	German shepherd	4	nasal cavity	H	Khenchela
PAE_F20	dog	M	Poodle	8	middle ear	H	Khenchela
PAE_CN14	dog	M	na	na	rectum	H	Batna
PAE_28AN	dog	F	Crossbred	48	nasal cavity	H	Setif
PAE_ESP	cat	na	na	na	rectum	na	Batna
PAE_173	cat	na	na	na	rectum	H	Batna
PAE_207	cat	F	na	na	rectum	D (gastroenteritis)	Batna
PAE_204	cat	M	na	na	rectum	H	Batna

Abbreviations: na = not available; D = disease; H = healthy.

**Table 2:** Prevalence (%) of phenotypic antimicrobial resistance.

	Dogs		Cats		*p*-Value
Antibiotic	R	S	R	S	
Amoxicillin–clavulanic acid (AMC)	100 (15)	0	100 (4)	0	-
Ticarcillin (TC)	40 (6)	60 (9)	25 (1)	75 (3)	0.1
Ticarcillin–clavulanic acid (TCC)	13.33 (2)	87 (13)	25 (1)	75 (3)	0.530
Cefepim (FEP)	0	100 (15)	0	100 (4)	-
Ceftazidime (CAZ)	0	100 (15)	0	100 (4)	-
Aztreonam (ATM)	6.6 (1)	60	0	75 (3)	1
Imipenem (IMP)	20 (3)	67	75 (3)	25 (1)	0.303
Levofloxacin (LEV)	0	100 (15)	0	75 (3)	0.2
Ciprofloxacin (CIP)	0	100 (15)	0	100 (4)	-
Netilmicin (NET)	0	100 (15)	0	100 (4)	-
Tobramicin (TOB)	0	100 (15)	0	100 (4)	-
Gentamicin (CN)	0	100 (15)	0	100 (4)	-
Amikacin (AK)	0	100 (15)	0	75 (3)	0.211

**Table 3:** Results of nanopore sequencing and accession numbers of *P. aeruginosa* sequences.

ID	Source	BioSample Accession	Genome Accession	Comp	Cont	Cov	Contig N50 (bp)	Genome Size (bp)	GC (%)	Contigs	Assembly Level
PAE_16AN	Dog	SAMN43392783	CP169763	98.28	1.84	186	6,532,852	6,532,852	66	1	Chromosome
PAE_173	Cat	SAMN43392795	CP169759	96.06	2.29	151	6,605,328	6,605,328	66	1	Chromosome
PAE_204	Cat	SAMN43392797	CP169760	90.89	4.27	116	6,366,466	6,366,466	66	1	Chromosome
PAE_207	Cat	SAMN43392796	CP169762	93.97	2.5	114	6,602,785	6,602,785	66	1	Chromosome
PAE_28AN	Dog	SAMN43392793	JBHGZY000000000	96.02	1.64	161	6,594,872	6,661,963	66	2	Contig
PAE_32AN	Dog	SAMN43392779	CP169765	99.94	0.46	63	6,465,512	6,465,512	66	1	Chromosome
PAE_a2	Dog	SAMN43392790	CP169758	94.75	2.82	86	6,443,410	6,443,410	66	1	Chromosome
PAE_a3	Dog	SAMN43392782	JBHHAE000000000	95.94	0.95	92	4,124,627	6,542,771	66	5	Contig
PAE_CN10	Dog	SAMN43392780	JBHHAG000000000	99.35	1.69	70	6,314,619	6,410,152	66	3	Contig
PAE_CN14	Dog	SAMN43392792	JBHGZX000000000	92.72	1.4	178	6,593,826	6,691,624	66	3	Contig
PAE_CN17	Dog	SAMN43392789	JBHHAC000000000	93.18	3.98	148	6,510,536	6,570,451	66	2	Contig
PAE_CN5	Dog	SAMN43392784	CP169761	93.96	1.27	91	6,430,499	6,430,499	66	1	Chromosome
PAE_CN9	Dog	SAMN43392781	JBHHAF000000000	90.65	3.41	38	6,365,670	6,382,583	66	2	Contig
PAE_ESP	Cat	SAMN43392794	JBHHAD000000000	99.57	2.1	171	6,755,612	7,332,217	66	3	Contig
PAE_F20	Dog	SAMN43392791	JBHHAB000000000	95.06	2.26	136	6,596,684	6,701,622	66	2	Contig
PAE_GAMMA	Dog	SAMN43392785	JBHHAA000000000	95.31	1.79	136	6,358,597	6,483,109	66	2	Contig
PAE_Q3	Dog	SAMN43392788	JBHGZW000000000	93.33	0.75	133	3,680,952	6,608,695	66	5	Contig
PAE_Q4	Dog	SAMN43392787	JBHGZZ000000000	94.82	1.78	137	6,564,132	6,615,011	66	2	Contig
PAE_SAV5	Dog	SAMN43392786	CP169764	95.11	1.52	78	6,211,070	6,211,070	66	1	Chromosome

Abbreviations: Comp = completeness; Cont = contamination; Cov = coverage.

## Data Availability

The strain descriptions and accession numbers are presented in Table 3; the genome assemblies, genomic data and raw data are publicly available in GenBank under BioProject PRJNA1153397 and genome accession numbers CP169763, CP169759, CP169760, CP169762, JBHGZY000000000, CP169765, CP169758, JBHHAE000000000, JBHHAG000000000, JBHGZX000000000, JBHHAC000000000, CP169761, JBHHAF000000000, JBHHAD000000000, JBHHAB000000000, JBHHAA000000000, JBHGZW000000000, JBHGZZ000000000 and CP169764.

## Supplementary Materials

The following supporting information can be downloaded at: https://www.mdpi.com/article/10.3390/antibiotics14030230/s1, Table S1: Distribution of STs according to geographical area.
