# Supplementary material for: Outbreak of Carbapenem-Resistant High-Risk Clone ST244 of Pseudomonas aeruginosa in Dogs and Cats in Algeria
Source: Antibiotics (Basel). 2025 Feb 24;14(3):230. doi: 10.3390/antibiotics14030230 (PMC11939618; doi:10.3390/antibiotics14030230)
Supplement: Supplementary file 1 [file antibiotics-14-00230-s001.zip › antibiotics-3491970-supplementary.docx]

Table S1: Distribution of STs according to geographical area.

| Strain | Sequence type | Geographical location |
| --- | --- | --- |
| PAE_173 | [244](https://pubmlst.org/bigsdb?page=profileInfo&db=pubmlst_paeruginosa_seqdef&scheme_id=1&profile_id=244) | Batna |
| PAE_204 | [1247](https://pubmlst.org/bigsdb?page=profileInfo&db=pubmlst_paeruginosa_seqdef&scheme_id=1&profile_id=1247) |  |
| PAE_207 | [244](https://pubmlst.org/bigsdb?page=profileInfo&db=pubmlst_paeruginosa_seqdef&scheme_id=1&profile_id=244) |  |
| PAE_CN10 | [1248](https://pubmlst.org/bigsdb?page=profileInfo&db=pubmlst_paeruginosa_seqdef&scheme_id=1&profile_id=1248) |  |
| PAE_CN14 | [244](https://pubmlst.org/bigsdb?page=profileInfo&db=pubmlst_paeruginosa_seqdef&scheme_id=1&profile_id=244) |  |
| PAE_CN17 | [1415](https://pubmlst.org/bigsdb?page=profileInfo&db=pubmlst_paeruginosa_seqdef&scheme_id=1&profile_id=1415) |  |
| PAE_CN5 | [189](https://pubmlst.org/bigsdb?page=profileInfo&db=pubmlst_paeruginosa_seqdef&scheme_id=1&profile_id=189) |  |
| PAE_CN9 | [1247](https://pubmlst.org/bigsdb?page=profileInfo&db=pubmlst_paeruginosa_seqdef&scheme_id=1&profile_id=1247) |  |
| PAE_ESP | [1342](https://pubmlst.org/bigsdb?page=profileInfo&db=pubmlst_paeruginosa_seqdef&scheme_id=1&profile_id=1342) |  |
| PAE_SAV5 | [16](https://pubmlst.org/bigsdb?page=profileInfo&db=pubmlst_paeruginosa_seqdef&scheme_id=1&profile_id=16) |  |
| PAE_a2 | [388](https://pubmlst.org/bigsdb?page=profileInfo&db=pubmlst_paeruginosa_seqdef&scheme_id=1&profile_id=388) | Khenchela |
| PAE_a3 | [2788](https://pubmlst.org/bigsdb?page=profileInfo&db=pubmlst_paeruginosa_seqdef&scheme_id=1&profile_id=2788) |  |
| PAE_F20 | [244](https://pubmlst.org/bigsdb?page=profileInfo&db=pubmlst_paeruginosa_seqdef&scheme_id=1&profile_id=244) |  |
| PAE_Q3 | [244](https://pubmlst.org/bigsdb?page=profileInfo&db=pubmlst_paeruginosa_seqdef&scheme_id=1&profile_id=244) |  |
| PAE_Q4 | [244](https://pubmlst.org/bigsdb?page=profileInfo&db=pubmlst_paeruginosa_seqdef&scheme_id=1&profile_id=244) |  |
| PAE_GAMMA | [343](https://pubmlst.org/bigsdb?page=profileInfo&db=pubmlst_paeruginosa_seqdef&scheme_id=1&profile_id=343) | M’sila |
| PAE_28AN | [244](https://pubmlst.org/bigsdb?page=profileInfo&db=pubmlst_paeruginosa_seqdef&scheme_id=1&profile_id=244) | Setif |
| PAE_32AN | [4160](https://pubmlst.org/bigsdb?page=profileInfo&db=pubmlst_paeruginosa_seqdef&scheme_id=1&profile_id=4160) |  |
| PAE_16AN | [1722](https://pubmlst.org/bigsdb?page=profileInfo&db=pubmlst_paeruginosa_seqdef&scheme_id=1&profile_id=1722) |  |
